# Developmental profiles of infants with an *FMR1* premutation

**DOI:** 10.1186/s11689-016-9171-8

**Published:** 2016-11-03

**Authors:** Anne C. Wheeler, John Sideris, Randi Hagerman, Elizabeth Berry-Kravis, Flora Tassone, Donald B. Bailey

**Affiliations:** 1 RTI International, 3040 E. Cornwallis Road, P.O. Box 12194, Research Triangle Park, NC 27709 USA; 2 University of North Carolina at Chapel Hill, Chapel Hill, NC USA; 3 Davis MIND (Medical Investigation of Neurodevelopmental Disorders) Institute, University of California at Davis, Davis, CA USA; 4 Department of Pediatrics, University of California at Davis, Davis, CA USA; 5 Rush University Medical Center, Chicago, IL USA

**Keywords:** *FMR1* premutation, Early development, Newborn screening

## Abstract

**Background:**

Emerging evidence suggests that a subset of *FMR1* premutation carriers is at an increased risk for cognitive, emotional, and medical conditions. However, because the premutation is rarely diagnosed at birth, the early developmental trajectories of children with a premutation are not known.

**Methods:**

This exploratory study examined the cognitive, communication, and social-behavioral profiles of 26 infants with a premutation who were identified through participation in a newborn screening for fragile X syndrome pilot study. In this study, families whose newborn screened positive for an *FMR1* premutation were invited to participate in a longitudinal study of early development. Twenty-six infants with the premutation and 21 matched, screen-negative comparison babies were assessed using validated standardized measures at 6-month intervals starting as young as 3 months of age. The babies were assessed up to seven times over a 4-year period.

**Results:**

The premutation group was not statistically different from the comparison group on measures of cognitive development, adaptive behavior, temperament, or overall communication. However, the babies with the premutation had a significantly different developmental trajectory on measures of nonverbal communication and hyperresponsivity to sensory experiences. They also were significantly more hyporesponsive at all ages than the comparison group. Cytosine-guanine-guanine repeat length was linearly associated with overall cognitive development.

**Conclusions:**

These results suggest that infants with a premutation may present with subtle developmental differences as young as 12 months of age that may be early markers of later anxiety, social deficits, or other challenges thought to be experienced by a subset of carriers.

## Background

Individuals with premutation range cytosine-guanine-guanine (CGG) repeats (55–200) on the fragile X mental retardation (*FMR1*) gene were originally considered unaffected carriers whose primary health risk was the chance of offspring with fragile X syndrome (FXS). However, individuals with a premutation (“carriers”) are now known to be vulnerable to two later onset disorders—fragile X-associated tremor/ataxia syndrome (FXTAS) [1, 2] and fragile X-associated primary ovarian insufficiency (FXPOI) [3], and may also be vulnerable to multiple other medical, emotional, and cognitive challenges [4]. Population studies suggest a high prevalence of the premutation, with as many as 1:150 women and 1:450 men estimated to be carriers [5–8]. This increased risk of health concerns and high population prevalence has led to a growing interest in the premutation phenotype [9–12]. However, most research on the premutation has focused on adults. Studies of children or adolescents nearly all involve participants who were identified because of the diagnosis of a close family member with FXS, biasing the sample toward increased likelihood of challenges.

Consistent with referral bias, studies of children with the premutation who were clinically referred (probands) suggest increased rates of developmental problems compared to noncarrier siblings [13–15] and carriers who were nonprobands—identified through cascade testing [16–18]. Further, increased risk for nonspecific developmental delay among children with premutation has been reported [19]. However, other studies report intact cognitive abilities in adults with a premutation *without* FXTAS [20]. Subtle cognitive difficulties have been reported among adults, most notably in executive functioning [20, 21], working memory [22], visual-spatial perceptual deficits [11], arithmetic [23, 24], speech fluency [25], and pragmatic language [26].

Several studies have documented more attention regulation, anxiety, and autism-like symptoms among carriers compared to controls. Hunter and colleagues [27] found that adult female carriers self-reported significantly more symptoms of inattention, memory, and poor self-concept compared to controls, although they did not score lower on cognitive tests. Anxiety symptoms, including social avoidance, interpersonal sensitivity, shyness, and eye contact avoidance, have been described in carriers, with up to 41 % reporting a lifetime diagnosis of an anxiety disorder [28, 29]. A higher than expected rate of autism spectrum disorders (ASDs) have also been reported for carriers [16, 18, 19, 30], although all these studies had some level of bias. More subtle ASD symptoms, such as social aloofness [28], rigid perfectionism [31], and features of the broad autism phenotype, have been described among carrier women [26]. These findings, along with studies linking the protein encoded by *FMR1*—the fragile X mental retardation protein (FMRP)—to the regulation of several pathways associated with autism [32–35], suggest a possible link between *FMR1* and autism-related phenotypes.

Because the premutation is rarely diagnosed in infancy, only one study has examined early development. This study examined 14 infants with the premutation and found visual processing deficits similar to infants with FXS, although their overall developmental scores were higher than those with FXS [36]. The findings suggested that deficits in spatiotemporal processing and subsequent executive dysfunction in some adults with the premutation may be present very early in life and emphasize the importance of studies examining early development of individuals along the spectrum of *FMR1* mutations.

Here, we report findings from a longitudinal study of infants identified with a premutation in a pilot study of newborn screening for FXS. This is a unique sample as the babies were identified and initially assessed within the first few months of life. Further, nearly all families were naïve to FXS or the premutation prior to the birth of their child and therefore had no preconceived information regarding the development of children with a premutation, and none of the babies were clinically referred. We were primarily interested in the very early cognitive, communication, and social-emotional development of infants with the premutation compared with screen-negative comparison infants. Given studies of older children and adults with a premutation, we did not expect to find significant differences in cognitive trajectories; however, we hypothesized that a subsample of infants with the premutation would demonstrate developmental differences compared to infants without a premutation.

## Methods

### Procedures

With approval from the Institutional Review Board, families were recruited in the postpartum unit within 24 h of the birth of their child. Recruitment took place over 4 years at three university-affiliated hospitals. Details about recruitment procedures and laboratory assessments can be found in previously published reports [8, 37]. To be included, the mother had to be at least 15 years of age, fluent in either Spanish or English, not undergoing stressful medical or personal circumstances, and not have infants in critical care for life-threatening conditions or relinquished for adoption. Newborns with a premutation allele were identified using polymerase chain reaction (PCR) analysis as reported by Tassone et al. [8]. Because the study was intended as a pilot for newborn screening, the molecular data collected reflects only the information necessary to identify an *FMR1* mutation; additional molecular measures including activation ratio and methylation status were not assessed.

Out of the 12,709 infants screened across the three sites, one baby screened positive for the presence of a full mutation and 45 screened positive for the presence of a premutation. Families of screen-positive children were called by a medical geneticist or clinician specializing in FXS on the research team and offered a genetic counseling appointment and confirmatory testing. Sixteen families did not have confirmatory testing because they declined further participation (*n* = 3), did not show up for the diagnostic appointment (*n* = 2), declined repeat testing (*n* = 3), or were unable to be reached via phone or mail (*n* = 8).

All families whose babies had a confirmed expansion following the diagnostic testing were invited to join the longitudinal component of the study. Twenty-eight infants who screened positive for an *FMR1* mutation participated in at least one longitudinal assessment. The baby with FXS and one baby who also screened-positive for Klinefelter’s syndrome were not included in the analyses, resulting in a total of 26 infants. A set of comparison tests on demographic variables (maternal age, marital status, race/ethnicity, maternal education) and child CGG repeat length for families who participated in the longitudinal study and those who screened positive but declined participation was conducted, yielding no significant differences between participants and nonparticipants [38] (Table 1).

**Table 1:** Demographics of sample

	Premutation sample (*n* = 26)	Comparison sample (*n* = 21)
Number of assessments	76	48
Gender	15 females	15 females
	11 males	6 males
Ethnicity	14 (54 %) White	12 (57 %) White
	6 (23 %) African American	5 (24 %) African American
	2 (8 %) Hispanic	2 (10 %) Hispanic
	4 (15 %) other	2 (9 %) other
CGG repeat (range)	55–125	–

Following diagnostic confirmation, families who agreed to participate in the follow-up study were scheduled for their first assessment when the child was 6 months of age. Assessments were scheduled at approximately 6-month intervals. All assessments were conducted at the family’s home or at a hospital/clinic-based setting. Assessors were trained by a licensed psychologist on all measures. For most assessments, the assessors were not blind to diagnostic group; however, some measures were double-scored by measure experts who were blind to diagnostic grouping.

### Participants

Of the 26 infants with a premutation who participated in at least one assessment, 15 were female. Fifty-four percent of the sample was Caucasian, 23 % was African American, and 8 % was Hispanic. Two sets of twins both with a premutation were included. The range of CGG repeats in infants with the premutation was 55–125, with the majority of the premutation alleles below 70 CGG repeats (77 %).

Because participants entered the study at different times over the course of the 4-year enrollment period, as well as some attrition (*n* = 6), infants were assessed anywhere between one and seven times (see Table 2). Comparison tests, chi-square frequency tests for categorical variables and *t* tests for continuous variables, were run to examine potential differences in family demographic and child variables for families who stayed in the study versus those who dropped out after at least one data collection visit. We found no evidence for differences in gender, race, mother’s education, age, or CGG repeat of the infant (all *p* > .27).

**Table 2:** Number of assessments at each age point, by group

Group	3–4 months	5–7 months	11–12 months	16–19 months	22–25 months	29–31 months	35–42 months
Premutation	3	22	18	10	10	5	11
Comparison	0	15	10	8	6	5	4

All families who participated in the newborn screening pilot study provided basic demographic information and were told at the time of providing consent that they may be asked to participate in a longitudinal study as a comparison family should their infant screen negative. Comparison infants were recruited from the sample of babies who screened negative for any *FMR1* expansion if they were a match for a screen-positive baby based on location (i.e., state of study site), gender, race and ethnicity, maternal education, and family income.

### Measures

Eight well-validated, standardized measures were used to assess early cognitive development, language and communication, social-emotional, and sensory behaviors.

#### Early cognitive development

*The Mullen Scales of Early Learning* [39] was administered at each assessment. The Mullen is a standardized developmental test for children birth to 68 months, assessing multiple developmental domains including expressive and receptive language, gross and fine motor skills, and visual processing. The *Vineland Adaptive Behavior Scales, Second Edition* (VABS-II) [40] was also completed in an interview format with the primary caregiver at each assessment point to measure functional behavior. The VABS-II provides parent reported information on functional skills in the areas of daily living, communication, socialization, and motor. Standard scores for all domains, as well as overall composites, were used for analyses for these measures.

#### Early language and communication

In addition to the broad measures of expressive and receptive language derived from the Mullen Scales of Early Learning (MSEL) and VABS-II, measures of more subtle communication and language development were included in the battery. The parent-reported *MacArthur Communicative Development Inventories, Second Edition* [41] (CDI) were used to assess more detailed information regarding the infants’ word production and early communicative intent. The CDI consists of two inventories, each with two sections. The CDI/Words and Gestures Inventory is for infants between the ages of 8 and 16 months. The inventory’s word section, which has a 28-item list of phrases and a 396-word checklist, is used to assess the infant’s production and understanding of words and phrases. The gesture section covers 63 gestures for communication, play, imitation of parents and other adults, and activities with objects. The CDI/Words and Sentences Inventory is for toddlers between the ages of 16 and 30 months. The inventory’s word section assesses vocabulary using a 680-word checklist. The second part assesses the toddler’s use of possessives, plurals, and tenses and development of complex sentences. The Words and Gestures Inventory was used at 18, and 24 months, replacing the CDI/Words and Gestures Inventory used at 12 months.

The *Communication and Symbolic Behavior Scale* (CSBS) [42] was used to assess functional communication. The CSBS is a norm-referenced screening and evaluation tool that helps determine the communicative competence (use of eye gaze, gestures, sounds, words, understanding, and play) of children with a functional communication age between 6 and 24 months (chronological age from about 6 months to 6 years). The scale yields three composite scores—social, speech, and symbolic—and concurrent and predictive validity are well established [42]. The caregiver report form and the directly administered behavioral profile components of the CSBS were administered. All direct administrations were videotaped and double-scored by a speech language pathologist with expertise in CSBS administration and a trained research assistant. Both coders were blind to the diagnostic status of the participants.

#### Social-emotional and sensory behaviors

Due to reports of an increased incidence of autism, attention, and anxiety symptoms among older children and adults with the premutation, several measures of early social communication and sensory behaviors were included. The *Sensory Experiences Questionnaire* [43] was completed by the caregivers at each data collection point to assess behavioral responses to common sensory experiences (e.g., child dislikes cuddling). Thirty-three items are used to calculate (as simple means) a total score and subscales for hyperresponsiveness (14 items), hyporesponsiveness (6 items), and sensory seeking (13 items). The Sensory Experiences Questionnaire (SEQ) categorizes scores as “normal,” “at-risk,” or “deficient” based on the number of standard deviations from the mean score for the norming sample [44]. In addition, two measures of autism symptoms were administered. The *First Years Inventory* (FYI) [45] was completed with participants assessed at 12 months. The FYI was developed to assess the presence of behaviors at 12 months associated with the later development of an autism spectrum disorder. Finally, for those whose last assessment occurred after 24 months, the second edition of the *Autism Diagnostic Observation Schedule-2* [46] (ADOS-2) was administered.

### Data analysis

Correlations between CGG repeat length and all variables were examined for those in the premutation group, as were comparison (*t* tests of continuous variables; chi-square for categorical) tests between males and females. For these analyses, we chose the assessment point at the oldest age for each child. For comparison tests between groups, standard scores on all measures were averaged for each participant across all assessments they completed. Despite the small *n*, longitudinal data analyses were collected at up to seven time points per child for a total of 124 assessments, allowing us to run hierarchical linear models (HLM) on selected variables. Given the large number of potential measures, we selected variables for HLM based on two criteria: (1) variables related to potential differences reported in older individuals with a premutation (social, communication, sensory) and (2) variables demonstrating some divergent patterns upon preliminary descriptive analysis. The primary hypotheses under examination focused on the relationship of child age and diagnostic group to the outcomes of interest. We tested for nonlinear (quadratic) change over time, but there was no evidence that such trends existed, so all models were simplified to include only linear terms. We treated the models for the child outcomes as two-level hierarchical linear models where time is nested within child. Repeated observations of subjects introduce dependence in the data. HLM manages this through the estimation of random effects in addition to traditional regression parameters [47]. The random effects provide estimates for within subject variance to control for this dependence. These models included random effects for the intercept. In all models, age and the moderators were grand mean centered, so any group differences are at the mean of those variables. The data are coded so that the premutation group is the reference. The group parameters in Table 4, then, provide the mean difference between the groups; positive parameters indicate a higher score in the comparison group and negative parameters indicate a higher score in the treatment group. The parameter for age represents the change over time for the comparison group. The interaction indicates how much the premutation group deviated from that rate. The absence of an interaction indicates that the mean difference is constant across all ages and the range of the moderator. Where interactions were suggested, graphs were generated to illustrate the findings.

Our small sample size provided a substantial barrier to our statistical inferences. Basic statistical theory suggests that parameter estimates in samples of this size are likely to be unreliable (e.g., Gravetter and Wallnau [48, p. 204]). Further, standard error is inversely related to sample size, so power decreases as sample size decreases. Having repeated measurement and testing in HLM framework provides an increase to statistical power [49]. We produced regression diagnostics (e.g., residual and normality plots) for all of the statistical models in the analysis. Examination of these plots indicated that normality and homoscedasticity assumptions were met and provided no suggestion of outliers.

The difficulties arising from the small sample size are compounded by the relatively large number of dependent variables. Typically, the possibility of false discovery as a function of multiple testing leads researchers to make adjustments to the critical values or *p* values for the results (see Benjamini and Hochberg [50] for a complete discussion). We opted to make no adjustments, however. Adjusting for multiple testing is intended to control for type I error. Given the exploratory nature of this study, we suggest that the uniqueness of this sample and the potential for these results to provide useful effect size estimates for future research makes type II error of greater concern, but readers should keep the possibility of false discovery in mind.

## Results

### Developmental profiles

Average scores on the Mullen across diagnostic groups were similar over time, with mean scores consistently in the low average to average range for both groups. There were no significant differences between diagnostic groups on any of the MSEL domains (expressive language, receptive language, fine motor, gross motor, visual reception) at any time point. Parent-reported adaptive behaviors measured by the VABS-II composite and domain scores were age appropriate for both groups across all age points. There was no significant age-by-group interaction for any MSEL or VABS-II domain. However, CGG repeat length was negatively correlated with overall (last administered) MSEL scores (−.39; *p* = .05); children with higher repeats had lower overall development. See Table 3 for mean scores across all assessment points by group on the MSEL and VABS-II.

**Table 3:** Means and standard deviations on key measures across all assessment points

Assessment	Premutation sample: mean (SD)	Comparison sample: mean (SD)	*p* value for test
Mullen ELC^a^	89.1 (14.6)	92.8 (17.7)	.436
Mullen GM^b^	48.7 (11.0)	49.2 (14.3)	.896
Mullen FM	46.0 (10.9)	49.5 (11.2)	.287
Mullen VR	44.3 (11.4)	47.9 (12.0)	.301
Mullen RL	44.9 (11.7)	45.0 (13.3)	.979
Mullen EL	41.8 (10.2)	42.1 (12.3)	.929
VABS-II ABC^c^	97.9 (10.7)	100.3 (9.0)	.408
VABS-II communication	98.1 (12.3)	100.4 (8.1)	.446
VABS-II daily living skills	100.5 (10.9)	100.5 (13.9)	1.00
VABS-II motor	98.0 (14.5)	100.1 (10.2)	.564
VABS-II socialization	97.3 (8.7)	101.1 (7.4)	.113
SEQ hyporesponsiveness^d^	11.6 (9.3)	9.3 (2.0)	.226
SEQ hyperresponsiveness	24.9 (5.6)	24.4 (4.3)	.730
SEQ seeking*	32.8 (9.9)	22.2 (12.5)	.003
CSBS-parent report total^e^	96.7 (14.9)	98.5 (14.0)	.672
CSBS-parent emotion and eye gaze^f^	10.9 (3.12)	11.3 (2.23)	.611
CBSB-parent-communication	10.9 (4.57)	11.3 (2.92)	.718
CSBS-parent-gestures*	11.2 (3.98)	13.6 (4.12)	.050
CSBS-parent-sounds	9.6 (3.92)	11.0 (3.75)	.219
CSBS-parent-words	11.2 (3.45)	11.1 (2.93)	.915
CSBS-parent-understanding	9.0 (3.33)	9.7 (3.42)	.484
CSBS-parent-object use	9.7 (3.34)	9.4 (2.75)	.737
CSBS-parent-social	10.5 (3.29)	11.1 (2.77)	.500
CSBS-parent-speech	9.5 (3.40)	9.9 (3.42)	.691
CSBS-parent_symbolic	8.9 (2.71)	9.1 (2.98)	.813
CSBS-direct total	88.3 (12.7)	89.9 (10.0)	.631
CSBS-direct-emotion and eye gaze*	8.8 (2.59)	10.7 (3.61)	.049
CBSB-direct-communication	8.8 (3.27)	8.7 (2.13)	.900
CSBS-direct-gestures*	8.3 (2.52)	10.0 (2.00)	.013
CSBS-direct-sounds	8.3 (1.89)	9.1 (2.17)	.190
CSBS-direct-words*	10.3 (2.50)	8.9 (2.05)	.041
CSBS-direct-understanding	9.1 (3.00)	7.9 (2.22)	.122
CSBS-direct-object use	7.4 (2.63)	7.2 (2.96)	.809
CSBS-direct-social	8.4 (2.52)	9.6 (1.80)	.064
CSBS-direct-speech	8.8 (2.60)	8.9 (2.00)	.882
CSBS-direct-symbolic	7.4 (2.17)	7.1 (2.59)	.674

^a^Mullen ELC = Early Learning Composite; mean = 100, SD = 15

^b^Mullen GM = gross motor; FM = fine motor; VR = visual reception; RL = receptive language; EL = expressive language; mean = 50, SD = 10

^c^VABS-II ABC = Vineland Adaptive Behavior Composite; mean for all VABS-II scales = 100, SD = 15

^d^SEQ = Sensory Experiences Questionnaire; hyporesponsiveness reference mean = 8.7 (1.9); hyperresponsiveness reference mean = 24.1 (4.6); seeking reference mean = 29.7 (8.7) [76]

^e^CSBS total = Communication Symbolic Behavior Scale; mean = 100, SD = 15

^f^CSBS subscales (emotion and eye gaze, communication, gestures, sounds, words, understanding, object use, social, speech, symbolic); mean = 10, SD = 3

*significant difference between groups

### Language profiles

**Fig. 1:**
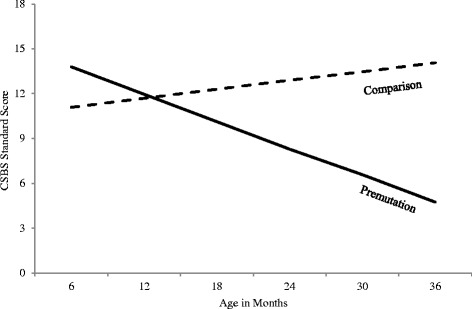
CSBS parent report emotion and eye gaze scaled scores by age and diagnosis

There were no differences between gender or diagnostic groups at any age point on any of the CDI variables. Scores on the parent report and the direct administered CSBS suggested some differences between the groups however. For the parent report version, across all time points, babies with the premutation were reported to display fewer gestures than the screen-negative babies. On the direct assessment measure, babies with the premutation were rated as having fewer gestures, poorer emotion and eye gaze, and more use of words than the comparison babies (see Table 3). There was a significant interaction between age and group status for the emotion and eye gaze subscale (see Table 4), which measures early social-emotional and nonverbal communication behaviors. While the comparison infants gained skills on this subscale over time, eventually reaching the ceiling by 36 months, babies with the premutation did not show the same increases in skills, resulting in decreasing scaled scores over time (see Fig. 1 and Table 3).

**Table 4:** Parameter estimates and (standard errors) for early development (Mullen, VABS-II), communication (CSBS), and sensory issues (SEQ hyper, hypo, and seeking)

Effect	Intercept	Group	Age	Group × age
Mullen ELC	98.43 (2.88)	4.31 (4.27)	.12 (.15)	−.02 (.25)
VABS-II ABC	98.11 (2.05)	1.88 (2.96)	.11 (.11)	.13 (.19)
CSBS-emotion and eye gaze	10.92 (.77)	1.12 (1.14)	−.31 (.09)**	.41 (.16)*
SEQ-hyporesponsive	10.96 (.50)	−1.51 (.79)*	.02 (.04)	.00 (.07)
SEQ-hyperresponsive	24.96 (.66)	−.93 (1.06)	−.11 (.05)*	−.21 (.09)*
SEQ-seeking	31.29 (1.32)	1.64 (2.11)	−.25 (.1)	−.28 (.07)

*p < .05; **p < .01

### Behavioral profiles

**Fig. 2:**
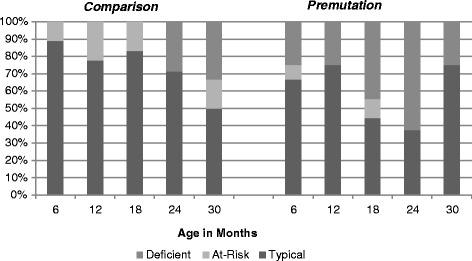
Prevalence of hyporesponsiveness scores across SEQ defined categories

**Fig. 3:**
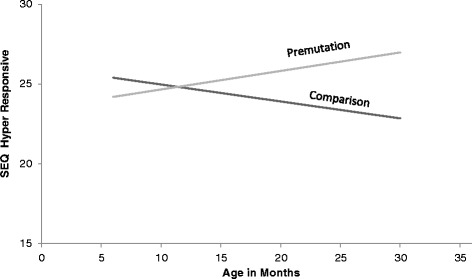
SEQ hyperresponsive scores across time, by group

There were no significant differences between males and females with a premutation on any of the behavior domains. However, there were significant differences between diagnostic groups across all assessment points on sensory seeking, with the babies with the premutation displaying more sensory-seeking behaviors. There were also significant differences between groups over time on both hypo- and hyperresponsivity on the SEQ. There were significant main effects for hyporesponsiveness, with the premutation group more hyporesponsive at every age point assessed (see Fig. 2). There was also a significant interaction effect for hyperresponsiveness (see Table 4); while the comparison group became less hyperresponsive as they got older (a normative pattern), the premutation babies became more hyperresponsive over time (see Fig. 3).

### Autism symptoms

There were no significant differences between groups on any of the scales on the FYI. However, while none of the comparison babies and just 4 % of the norming sample for the FYI [51] scored above 98th percentile on the total risk variable, almost a third of the premutation babies (27 %) scored in this range. Although there were too few participants who completed an ADOS-2 to compare statistically across groups, three out of the eight premutation babies who were tested scored in the elevated range, compared to none of the controls. There were no gender differences on these measures.

## Discussion

This exploratory study is the first to examine early developmental profiles of infants with an *FMR1* premutation. Amid emerging evidence that premutation CGG expansions on the *FMR1* gene are associated with increased risks for social, emotional, and medical difficulties, understanding the early trajectories of these risks is critical. We found that patterns of broad early development did not differ significantly from matched screened-negative babies. This was true for all of the areas of development traditionally assessed by pediatricians during well-baby visits. However, we found significant differences in several important variables related to social and sensory experiences as early as 12 months of age in premutation babies. Infants with the premutation had increased risk for social communication differences as well as both hypo- and hyperresponsiveness to stimuli when compared to both the comparison group, as well as norming samples. These differences suggest a profile of very early regulation challenges which may be related to increased risk for attention, anxiety, or autism symptoms reported for older individuals.

Infants with the premutation were found across all assessment points to be more sensory seeking than their matched screen-negative peers. They were also rated to be more hyporesponsive to sensory stimuli at all ages tested. Hyporesponsivity refers to a lack of or reduced intensity in response to external stimuli. For example, a hyporesponsive infant may not react to loud sounds or may appear to have a very high pain threshold. Patterns of increased hyporesponsivity have been associated with increased risks for autism in both toddlers [52] and older children [53, 54]. Specifically, hyporesponsivity is more strongly associated with the core autism symptoms of social and communication impairments than with repetitive behaviors [55], which are generally more related to hyperresponsivity [56]. Interestingly, the premutation group also became more hyperresponsive over time, whereas the control group became less hyperresponsive as they got older. Hyperresponsivity is used to describe a pattern of exaggerated responses to sensory stimuli—for example avoiding touch or covering ears to block sounds. Individuals with FXS are often described as being hyporesponsive in the first year of life and then become hyperresponsive and hyperaroused in response to external stimuli after the second or third year of life [57]. The hyperresponsive behavior is thought to be related to a lack of GABA inhibition which causes a lack of habituation to sensory stimuli seen in children with FXS [58]. GABA deficits have not only been documented in FXS but also in those with the premutation [59].

This pattern of both hyporesponsiveness and increasing hyperresponsiveness may appear counterintuitive; however, some literature suggests that this co-existing pattern is more common among children with developmental differences, especially autism symptoms [43, 57, 60]. Although it is not uncommon to observe a pattern of co-occurring hyper- and hyporesponsivity in children with developmental differences, other studies have noted that hyperresponsivity may be more strongly related to mental age [53], while hyporesponsivity is more strongly related to core autism features [43], which may explain the co-occurrence in very young cohorts. However, according to the dynamic theory of sensory processing [61], individual responses to stimuli and experiences are partially influenced by two thresholds—one related to orienting to stimuli (orienting threshold) and one associated with tolerance for stimuli (aversion threshold). When in the optimal band of stimulation between orienting and aversion thresholds, an individual is the most capable of attending and engaging. The wider the band between these two thresholds, the greater the person is at adapting to their environment. A hyporesponsive individual will have a higher threshold for orienting (i.e., require more intense input from a stimulus to perceive and attend). Conversely, a hyperresponsive person will have a lower threshold for aversion (i.e., require less intense stimulus to become averse). An individual who is both hypo- and hyperresponsive therefore will have a narrower optimal band of stimulation from which to engage with their world [62]. This pattern may affect the individual’s ability to communicate and interact appropriately with others, leading to increases in anxiety, attention, or autism-like symptoms.

Babies with the premutation also demonstrated elevated social-communication problems as measured by the parent reported CSBS (fewer gestures) and the CSBS direct assessment (fewer gestures, worse emotion and eye gaze). Further, consistent with studies of older individuals with the premutation, just over a third of infants in this study had social-communication difficulties as measured by the parent-reported FYI and the direct assessment on the ADOS-2. The FYI was developed specifically to assess the presence of behaviors at 12 months associated with the later development of an ASD [45]. However, it is also a sensitive tool for identifying early non-ASD developmental differences [63]. Therefore, while elevated scores do not suggest a clear trajectory toward an autism diagnosis, they do suggest increased risk for social communication challenges or other developmental delays.

Gender differences are generally expected given the *FMR1* is located on the X chromosome and females, with two X chromosomes, can have a more variable phenotype based on the X-activation ratio (percent of cells with the normal allele on the active X chromosome). We did not find any gender differences on any of our variables of interest. Unfortunately, we did not have additional molecular data (e.g., activation ratio), which could be an important addition to understanding potential gender differences among premutation carriers.

Why some individuals with the premutation develop cognitive, psychiatric, or medical problems and others do not is a key question for *FMR1* researchers. In the current sample, higher CGG repeats in the infants were associated with lower developmental scores on the Mullen but not on any of the variables where there was a significant difference from the comparison group. CGG repeat length has been implicated in the onset and severity of several associated features, although the relationship is still unclear. Higher CGG repeats in the premutation range are associated with lower levels of FMRP [64, 65], as well as increases in *FMR1* messenger ribonucleic acid (mRNA) levels [66, 67], both of which disrupt normal neuronal functioning. However, while some studies suggest a linear association, particularly for neurological problems (e.g., higher CGG repeats associated with more problems) [22, 66, 68], others have found a nonlinear association (particularly for ovarian or psychiatric problems) with greater severity among those with a CGG repeat number in the mid-premutation range [29, 69–74]. Additional genetic abnormalities (copy number variation) have been found in 20 % of carriers who have ASD or neurological problems [75], and these may have an additive effect, leading to a more severe phenotype. Another possibility is that carriers have a genetic susceptibility to stress [73] which may result in greater impact of every day stressors on the developing brains of these infants. These studies suggest that both molecular and gene-by-environment interaction studies are needed to fully understand who, among premutation carriers, is at greatest risk.

There are several limitations to this study and associated alternative explanations for our findings. Although larger than any other study examining infants with an *FMR1* premutation, our findings are still limited by the small sample size. We were underpowered for some of our analyses, especially with the decreasing number of control subjects in older ages. The small sample size and large number of variables does increase the chances for false discoveries, and this is an important limitation to keep in mind. However, in the areas where significant differences were found, the comparison sample tracked along with what would be expected based on norming samples for the instruments, whereas significant differences were observed for the premutation sample. Further, the variation in developmental trajectories found in the premutation infants is not inconsistent with findings for older individuals with a premutation, where developmental differences, especially in social and executive function domains, have been reported in a subsample [16, 18, 19, 30]. Therefore, we argue that our findings from this relatively large, unbiased sample of premutation infants establish an important basis for examining potential molecular and environmental factors which may influence outcomes for individuals with a premutation very early in life.

Although all screen-positive families from the newborn screening study were given the same opportunity to participate, it is possible that more families who had concerns about their child were willing to participate in the study over time, thereby reducing generalizability of the results. However, in our experience, more dysfunctional families tended not to want to participate initially, perhaps biasing the study to higher functioning children. Further, although we were obviously not able to follow the development of those who did not participate, there were no significant differences in family- or child-level variables of those who did not continue in the study after their first assessment and those who were followed for a longer period of time.

In addition, some significant results may be due to parents of the premutation babies being more sensitive to subtle differences as a result of increased anxiety on their part as to the impact of the premutation. However, parents did not report significant differences on any of the broad areas of development or temperament traits. Rather, differences were found in areas that might be expected for children with early regulation difficulties associated with later anxiety, hyperactivity, and other social-emotional challenges. Further, we found significant differences between groups on the direct assessment of the CSBS, which was scored by experts blind to diagnostic group. Finally, these are the same differences commonly reported among larger samples of older individuals with a premutation, suggesting there may be increased risks for some individuals with a premutation which begin very early in life.

## Conclusions

This study is the first to report on developmental trajectories of infants and toddlers with an *FMR1* premutation and no family history of FXS. Results from this study support the emerging discussion of an endophenotype for individuals on the spectrum of *FMR1* involvement—with increased risk for a subset of premutation carriers. Additional genotype-phenotype studies are needed to help identify which *FMR1* premutation carriers are at greater risk in order to inform the development of early, supportive interventions to reduce negative outcomes.

## Abbreviations

ADOS: Autism Diagnostic Observation Schedule
ASD: Autism spectrum disorder
CDI: MacArthur Communicative Development Inventories
CGG: Cytosine-guanine-guanine
CSBS: Communication Symbolic Behavior Scale
FMR1: Fragile X mental retardation gene
FMRP: Fragile X mental retardation protein
FXPOI: Fragile X premature ovarian insufficiency
FXS: Fragile X syndrome
FXTAS: Fragile X tremor/ataxia syndrome
FYI: First Years Inventory
HLM: Hierarchical linear modeling
mRNA: Messenger ribonucleic acid
MSEL: Mullen Scales of Early Learning
PCR: Polymerase chain reaction
SEQ: Sensory Experiences Questionnaire
VABS: Vineland Adaptive Behavior Scales
